# A not-so-sweet reactive mucocutaneous eruption

**DOI:** 10.1016/j.jdcr.2026.09.005

**Published:** 2026-09-10

**Authors:** Emely Tejeda, Elisa M. Schunkert, Cynthia X. Chan, Bijal Amin, Benedict Wu

**Affiliations:** a Albert Einstein College of Medicine, Bronx, New York; b Division of Dermatology, Montefiore Medical Center/Albert Einstein College of Medicine, Bronx, New York; c Department of Pathology, Montefiore Medical Center/Albert Einstein College of Medicine, Bronx, New York

**Keywords:** bullous erythema multiforme, bullous Sweet syndrome, erythema multiforme, Herpes simplex virus, HSV, neutrophilic dermatosis, Sweet syndrome

## Case

A 45-year-old woman with a history of migraines presented to the hospital with a 2-day history of a worsening pruritic and burning rash, accompanied by painful oral mucosal lesions. She reported no new medications. Review of systems was notable for myalgias and nasal congestion. Of note, the patient reported experiencing similar eruptions twice in the past 2 years, with the current eruption being the most extensive.

On physical examination, erythematous red to violaceous edematous pseudovesicular papules and plaques were observed, favoring the bilateral upper extremities’ extensor surfaces (Figs 1, *A* and 2, *A*) and bilateral knees (Fig 2, *A*). A few small vesicles were noted on the palms (Fig 2, *B*). The left buttock had a cluster of crusted erosions with scalloped borders (Fig 1, *C*), differing in morphology from the other papules and plaques. Oral examination revealed erosions of the hard palate.

**Fig 1:**
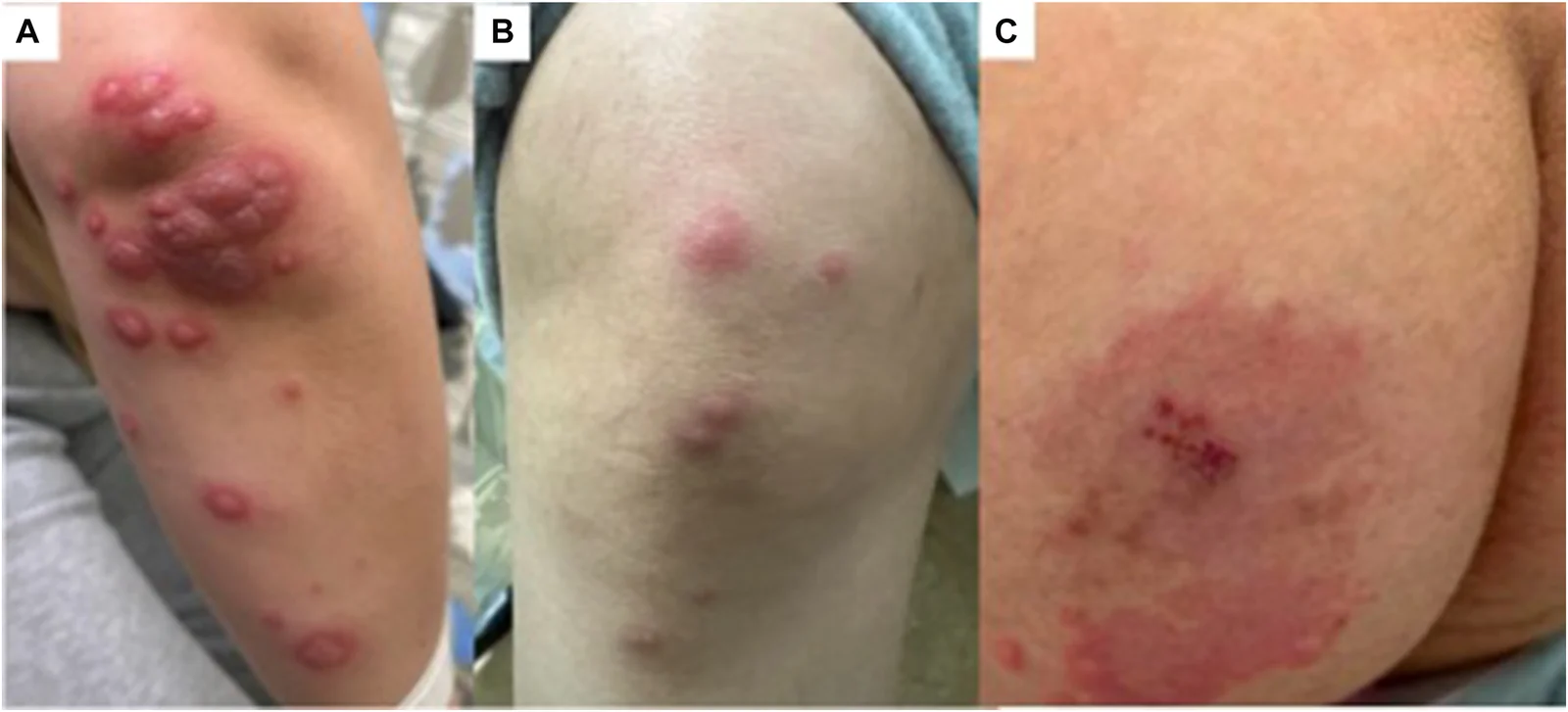
Red to violaceous, juicy, bullous, edematous, and pseudovesicular plaques and papules on the extensor surface of the right arm **(A)** and the extensor surface of the right knee **(B)**. Cluster of crusted erosions with scalloped borders on the left buttock **(C)**.

**Fig 2:**
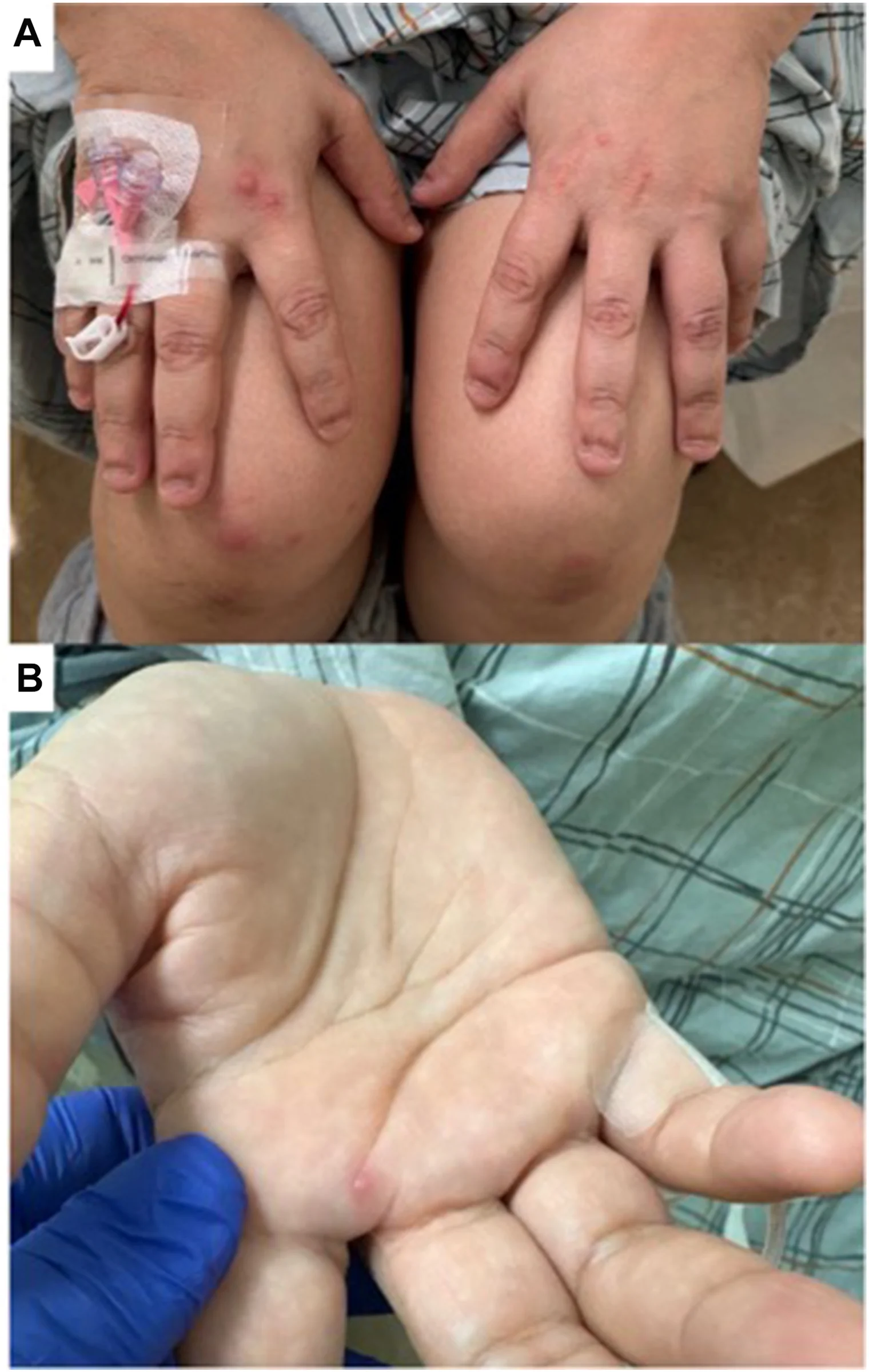
Pseudovesicular papules on an erythematous base on bilateral dorsal hands **(A)**, small vesicle located on the right palm **(B)**.

Herpes simplex virus (HSV) type-2 PCR testing from the left buttock returned positive. Pertinent negative tests were serum protein electrophoresis, anti-streptolysin antibodies, and rheumatoid factor. The skin biopsies from the right elbow showed purpuric interface dermatitis with vacuolar degeneration and necrotic keratinocytes in addition to numerous neutrophils (Figs 3 and 4); the direct immunofluorescence test and tissue culture were negative.

**Fig 3:**
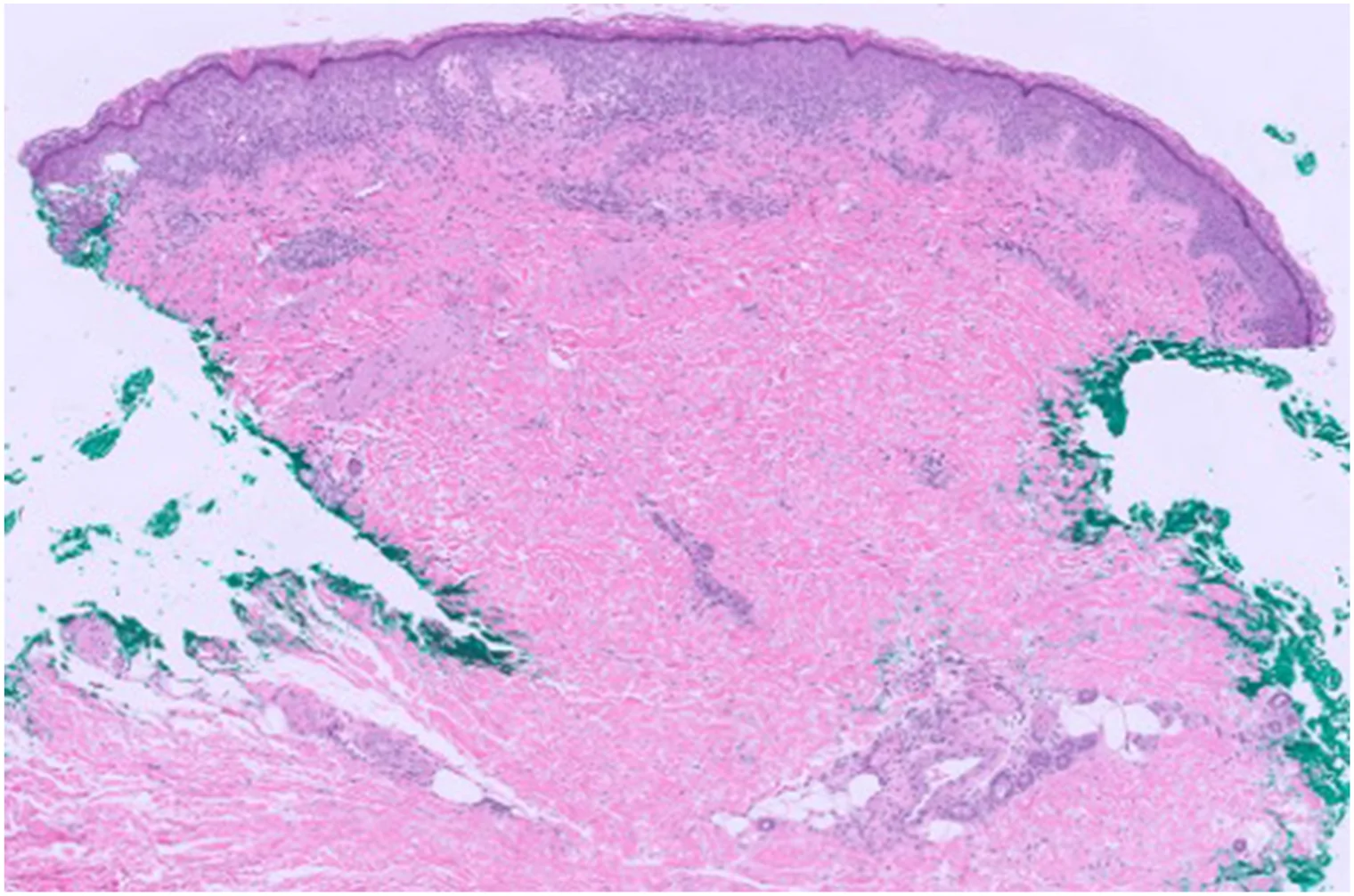
(H&E, 5×) A punch biopsy from the elbow showing purpuric interface dermatitis and necrotic keratinocytes.

**Fig 4:**
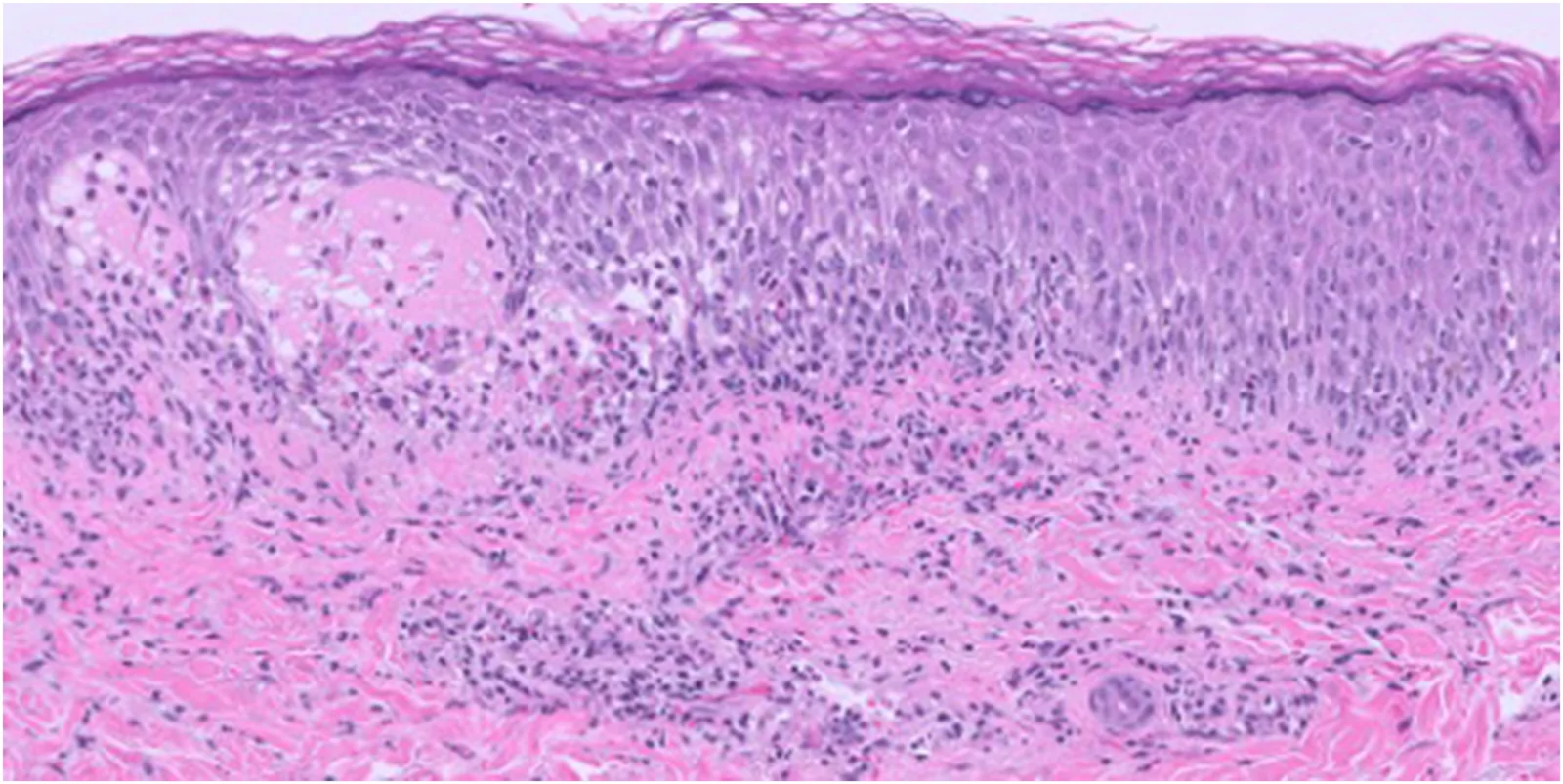
(H&E, 20×) Purpuric interface dermatitis with vacuolar degeneration and necrotic keratinocytes. Numerous neutrophils are also present within the inflammatory infiltrate.

****Question:** What is the most likely diagnosis?**

**A.** Fixed drug eruption
**B.** Sweet syndrome
**C.** Exaggerated arthropod bite reaction
**D.** Bullous erythema multiforme
**E.** Urticarial vasculitis

**Correct Answer: D. Bullous erythema multiforme.**

## Answer discussion

HSV is a common trigger for erythema multiforme (EM), with a subset of patients experiencing recurrent EM precipitated by recurrent HSV.^1^ This patient received oral valacyclovir and 40 mg of prednisone, tapered over 1 month. After 2 weeks, all her skin lesions had resolved, leaving only residual hyperpigmentation. The patient described recurrent EM episodes, possibly precipitated by recurrent HSV, with a favorable response to episodic therapy. However, suppressive therapy will be initiated should further episodes occur.

The presence of juicy papules and plaques masked the classic targetoid lesions, instead mimicking Sweet syndrome. Although the clinical morphology of EM lesions may vary, the characteristic distribution of the distal extremities, from the hands up to the elbows, and on the lower extremities up to the knees, is an important indicator and remains diagnostic of EM when supported by compatible clinical morphology and histopathology.

Bullous EM (BEM) is a rare variant of classic EM and has been reported to occur after exposure to tropical hardwood^2^ (Machaerium scleroxylon Tul.) and HSV/VZV.^3^ Interestingly, BEM is most often triggered by Mycoplasma pneumoniae and certain drugs,^4^ both of which were absent in our patient.

Prompt recognition of BEM is crucial, as these patients often require systemic corticosteroids, wound care, and hospitalization. Accurate diagnosis can be challenging, especially when lesion cultures are negative and biopsy results are indeterminate; our case showed neutrophil-predominant interface dermatitis, leading to the final diagnosis of BEM. Notably, only HSV-2 was detected, whereas HSV-1 is the more common culprit of EM.^5^

We present an interesting case of recurrent BEM due to HSV-2. Dermatologists should consider atypical presentations of EM in the differential diagnosis of suspected Sweet syndrome. Lastly, this case highlights the importance of clinico-pathologic correlation for proper diagnosis and management.

## Conflicts of interest

None disclosed.
